# A hemodynamic-driven approach to chronic pulmonary hypertension in congenital diaphragmatic hernia

**DOI:** 10.1038/s41372-026-02582-4

**Published:** 2026-02-23

**Authors:** Carly E. Byrd, John T. Wren, Suneetha Desiraju, Patrick J. McNamara

**Affiliations:** 1 Division of Neonatal-Perinatal Medicine, Department of Pediatrics, Johns Hopkins University School of Medicine, Baltimore, MD USA; 2 Division of Neonatology, Department of Pediatrics, University of Iowa Hospitals and Clinics, Iowa City, IA USA

**Keywords:** Respiration, Congenital heart defects

## Abstract

Despite ample research in acute pulmonary hypertension in neonates with congenital diaphragmatic hernia (CDH) leading to improved survival, there is a paucity of literature on the physiology of chronic pulmonary hypertension in this population. Serial hemodynamic assessments and physiology-centric care have been shown to be safe and effective in the acute transitional and peri-operative care of these neonates and will likely be informative in the discussion of chronic pulmonary hypertension as well. Herein, we discuss the epidemiology, contributors, and proposed physiology-based phenotypes of chronic pulmonary hypertension in neonates with CDH. Further investigation is needed to contribute to our understanding and treatment of chronic pulmonary hypertension in this complex, heterogeneous population.

## Introduction

Congenital diaphragmatic hernia (CDH) is a complex and multifactorial disease process that affects approximately 1 in 3000 neonates and encompasses a triad of overlapping yet distinct pathophysiologic conditions including lung hypoplasia, alterations in pulmonary blood flow (PBF), and cardiac dysfunction [1, 2]. While many studies have investigated the diagnosis and treatment of acute pulmonary hypertension (PH) in CDH, there is sparse literature on the pathophysiology, natural history, and treatment of *chronic* pulmonary hypertension (chronic PH) in the CDH population. Additionally, there is no widely established definition for chronic PH in this group. For this article, we propose the classification of chronic PH as persistent elevation in pulmonary artery pressure on echocardiography at least 3 months post-repair (selecting for those with prolonged hospitalizations and highest risk [3]) or at discharge, whichever occurs sooner. Inclusion must also include consideration of supplemental oxygen and PH medication requirements. There is a need for expert and multi-center consensus to establish a consistent definition of this disease process. Further, there must be a recognition of the risks of acute-on-chronic exacerbations within this population.

With advances in fetal interventions, lung-protective ventilation strategies, and limited use of inhaled nitric oxide only for select cases, survival rates of neonates with CDH have risen [4, 5]. Additionally, there is sparse literature on adult outcomes of infants born with CDH. With improving survival, an increasing number are diagnosed with chronic PH [6–8]. While there is a growing appreciation for the complex and multi-faceted physiology of acute PH in CDH, there are likely multiple contributors to chronic PH development as well [9].

Targeted neonatal echocardiography (TNE) and serial hemodynamic assessments are powerful tools that enable precision medicine in the neonatal intensive care unit [10–12]. TNE, in the acute management of neonates with CDH, has been shown to be both safe and effective [13, 14]. Beyond the acute pre- and peri-operative period, however, these neonates remain at high risk for the development of chronic PH during both their convalescent phase of care and post-discharge. While traditional TNE is limited to the initial inpatient setting [15], a similar multiparametric, physiology-driven echocardiogram in the outpatient setting also offers the capability to provide a comprehensive hemodynamic assessment regarding the development and management of chronic PH in the recovery period, post-discharge and into childhood.

Among the consensus CDH management guidelines, there is little discussion on the natural history, screening, and management of chronic PH in these patients [16, 17]. In this Perspective, we will appraise the use of comprehensive hemodynamic assessments in the diagnosis of chronic PH in CDH, discuss contributors to chronic PH development, propose clinical physiology-based phenotypes, and suggest an approach to chronic PH surveillance screening in this population.

## Chronic pulmonary hypertension in neonates with CDH: what is known

All neonates with CDH are at risk for developing chronic PH [6–8, 18–20]. While the incidence is likely low particularly after discharge, the true incidence rate is not definitively known. In a meta-analysis, Lewis and colleagues identified incidence rates ranging from 4.5 to 38% for the presence of PH in children with CDH at a mean age between 2 and 5 years [21] though selection bias is likely inflating the higher range of these numbers. In a large review of the Children’s Hospital’s Neonatal Consortium, Mahmood and colleagues identified an incidence of 11.6% of survivors requiring PH therapy at discharge [22]. Critser and colleagues recently found that 1 in 10 CDH survivors were found to have moderate-severe exercise-induced PH at a median age of 8 years although none had PH at rest, suggesting this may indicate persistent pulmonary vasoreactivity rather than traditionally defined PH [23]. This is also reflected by the gold standard of right heart cardiac catheterization where Zussman and colleagues found that children with CDH not only have higher mean pulmonary artery pressures (PAP) but also higher pulmonary vascular resistance (PVR) and reduced PBF at over a year of age compared to age-matched controls [20]. Risk factors further contribute to risk of chronic PH in CDH. Among those who required extracorporeal life support (ECLS), greater than one in three were diagnosed with chronic PH after discharge on echocardiography [6]. In the authors’ experience, 5-10% is likely a more accurate overall representation of the incidence; of note, this is likely to decrease with time although a definitive rate will remain unknown without an established PH screening regimen.

Long term outcomes are worse in the subset of CDH patients who also have chronic PH. This includes morbidities such as the need for supplemental oxygen and tube feedings and also includes mortality. Mortality rates are very low post-discharge in neonates with CDH but increased in those who progress to a diagnosis of chronic PH [1, 7, 8, 24]. With improved survival of CDH patients in the acute transitional and peri-operative period, it is imperative that we turn our attention towards the long-term care of these patients to optimize long term morbidity and mortality surrounding the development of chronic PH. Serial hemodynamic assessments afford this opportunity to optimize longitudinal care.

## Hemodynamic assessment of pulmonary artery pressure

Comprehensive, serial hemodynamic assessments are crucial in the management of patients with CDH. Combined TNE and consultation with fellowship-trained Neonatal Hemodynamics specialists, in accordance with the guidelines of the American Society of Echocardiography [15], has been used to provide echocardiography guidance in the *acute, peri-operative setting* to aid in diagnosing and treating acute PH in CDH [25]. Utilizing these same principles, serial echocardiography and physiology-based consultation has the potential to optimize care of PH in the s*ub-acute, post-operative*, and *post-discharge period*. A thorough understanding of multifactorial contributions to pulmonary pressures is critical [26].

Echocardiography estimates of PAP are derived from Ohm’s law of fluid hemodynamics which, when adapted for physiology, posits that the pressure difference across an organ is a function of the flow of blood into that organ multiplied by resistance (ΔP = Q x R). In the case of pulmonary vasculature, this (when re-arranged) becomes:

$$Pulmonary\,artery\,pressure\,(PAP)\,= 	\,(pulmonary\,vascular\,resistance\,[PVR]\,\\ 	\ \times \,pulmonary\,blood\,flow\,[PBF])\,\ \\ 	+\,left\,atrial\,pressure\,(LAp)$$

Here, we utilize PVR to refer specifically to pulmonary arterial vascular resistance, aligning with the gold standard diagnosis of PH by cardiac catheterization. While Ohm’s law represents a physiological categorization of elevated PA pressure, clinically PH is often categorized as “pre-capillary” or “post-capillary” PH largely in relation to their predicted response to pulmonary vasodilators^2^. In general, the former will correlate with elevated PVR and the latter with either (or both) elevated PBF and/or LA pressure, however, there are exceptions as will be discussed below.

A structured and systemic approach to estimating PAP is required [and described in detail for neonates with CDH elsewhere [27]]. In practice, this includes (in order of diagnostic significance) patent ductus arteriosus (PDA) shunt direction, tricuspid regurgitant (TR) jet to estimate right ventricular systolic pressure via the modified Bernoulli equation, and septal positioning. As the former is limited by PDA persistence (only one in three neonates with CDH have a PDA by post-natal day 30 [28]) and the second is restricted to the presence of a complete TR jet [29], the diagnosis of elevated PAP is often limited to the assessment of septal positioning. As the subjective assessment of septal position has poor interrater agreement [30], a quantitative measurement such as systolic eccentricity index is key [15].

Elevations in any of the three variables listed in the equation above, including PVR, PBF, or LA pressure, can all independently lead to PH, but will each present as a “flat septum” on echocardiography assessment. As we will discuss below, neonates with CDH are at risk for each of these physiologies. Thus, it is incumbent on the provider to incorporate a comprehensive hemodynamic assessment to isolate the relative contributions of each physiologic state to the underlying pathophysiology.

## Clinical phenotypes and contributors to chronic pulmonary hypertension in CDH

**Fig. 1:**
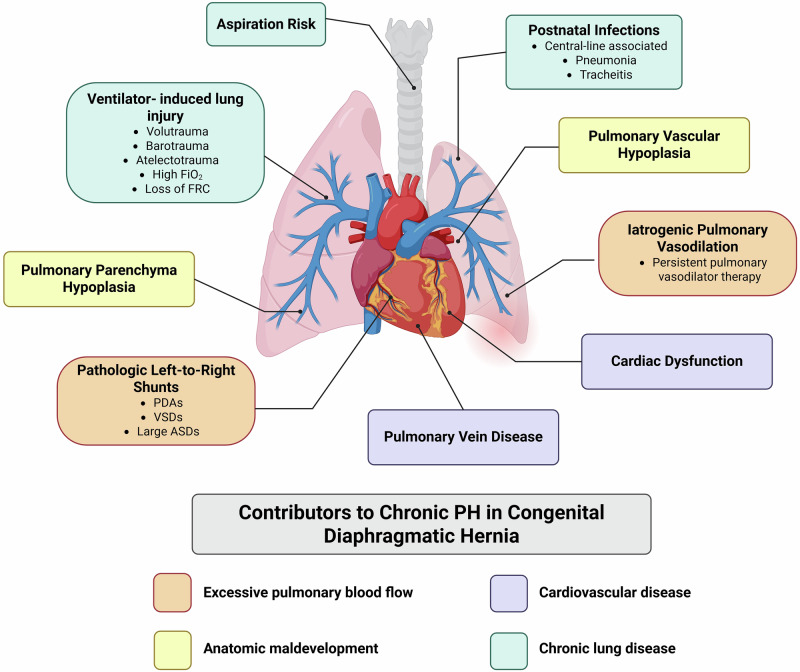
Contributors to chronic pulmonary hypertension in congenital diaphragmatic hernia. Components of chronic pulmonary hypertension development include anatomic maldevelopment, chronic lung disease, excessive pulmonary blood flow and cardiovascular disease.

As discussed above, disturbances in any variable of Ohm’s law (PBF, PVR or LA pressure) can cause elevated pulmonary pressures, and each entity has distinct physiologic mechanisms requiring different treatment strategies. Both the pulmonary parenchyma and vasculature are hypoplastic in all neonates with CDH, regardless of their hemodynamic phenotype, for multifactorial regions including *in utero* compression, reduced fetal blood flow across the foramen ovale, altered cell signaling molecules, and abnormal vascular development [31]. In addition, cardiac myocytes in neonates with CDH are intrinsically more susceptible to post-natal insults due in part to altered expression of key growth regulators including basic fibroblast growth factor and platelet derived growth factor [32]. Each of these can predispose neonates to the development of chronic PH. Risk factors for the development of chronic PH include[1]: the development of chronic lung disease from the use of volutrauma, barotrauma, atelectotrauma, and inflammation[2], excessive pulmonary blood flow[3], cardiac dysfunction and [4] aspiration and feeding related contributors (Summarized in Fig. 1). The relative contribution of each of these remains largely unknown. While *in utero* maldevelopment from defect size has often been favored as the predominant contributor, Mahmood and colleagues found post-natal variables (notably center, duration of mechanical ventilation, and duration of iNO) were most associated with PH medication need at discharge [22]. More research on primary drivers of PH in this population is needed. Below we describe each phenotype, their contributors, and postulated natural history in children with CDH (Table 1).

**Table 1:** Contributors, natural course and treatment of each clinical phenotype of chronic pulmonary hypertension in congenital diaphragmatic hernia (CDH).

Chronic PH Phenotype	Contributors	Natural course	Treatment modalities
Resistance-mediated Phenotype	-Lung hypoplasia -Pulmonary vascular hypoplasia -Ventilator induced lung injury -Infection -Aspiration	-Most common -Bimodal course ○ Initial PVR decline followed by increase after postnatal insults ○ Prolonged elevated PVR with postnatal morbidities	-Ventilator optimization ○ Optimal lung inflation ○ Target FRC and appropriate tidal volumes -Pulmonary vasodilator therapy (FiO_2_ or medications) -Prioritize somatic growth -Gastroesophageal reflux management
Flow-mediated Phenotype	- Iatrogenic pulmonary vasodilator use - Pathologic left-to-right shunts ○ PDAs, VSDs, ASDs	-Delayed ○ Occurs after persistent exposure to excess pulmonary blood flow	-Discontinuation of iatrogenic pulmonary vasodilators and prostaglandin infusions -Shunt mitigation strategies ○ Diuretics ○ Definitive shunt management
Post-capillary Phenotype	-LV diastolic dysfunction -Pulmonary vein disease -Aortic arch hypoplasia spectrum	-Varying depending on the underlying etiology -Further investigation needed	-Anti-hypertensive medications -Diuretics -Procedural intervention for pulmonary vein stenosis -Avoid pulmonary vasodilators
Mixed Phenotypes	-Flow mediated or post-capillary phenotype with development of resistance mediated phenotype	-Transition in physiology with components of multiple phenotypes	-Cautious, careful use of intermittent or low dose vasodilators and/or diuretics -Frequent echocardiographic re-assessments -Consider early cardiac catheterization to guide precision therapy

*PH* pulmonary hypertension, *PVR* pulmonary vascular resistance, *FiO2* Fraction of inspired oxygen, *FRC* functional residual capacity, *PDA* patent ductus arteriosus, *VSD* ventricular septal defect, *ASD* atrial septal defect, *LV* left ventricle.

### Resistance-mediated PH (PAP = PBF x PVR + LAp)

Resistance-mediated chronic PH is likely the most common chronic PH phenotype in CDH, as has similarly been seen in acute CDH PH [33]. This originates from a combination of intrinsic pulmonary vascular hypoplasia coupled with pathologic vascular remodeling and decreased vascular responsiveness to vasodilators [34]. Vascular remodeling occurs early in utero, but the majority likely occurs postnatally with the development of chronic lung disease, ventilator-induced lung injury, pathologic prolonged left to right shunt exposure, and aspiration.

Neonates with CDH are born with pulmonary hypoplasia which, along with other etiologies of the same condition, is intrinsically associated with pulmonary vascular hypoplasia secondary to embryonic maldevelopment and, in the case of CDH, extrinsic compression in utero [35] increasing the risk for chronic PH development [36, 37]. In addition, there is a growing appreciation of the “two-hit” hypothesis that includes a predisposing genetic mutation and/or polymorphism regarding pulmonary parenchymal and vascular disease [38]. Due to their pulmonary hypoplasia, these neonates operate at lower functional residual capacity (FRC) and require lower positive end expiratory pressures [39, 40]. Excessive ventilator settings aimed at ECLS avoidance can further exacerbate deviations from FRC with lung overdistension, volutrauma, and barotrauma contributing significantly to ventilator-induced lung injury [41, 42]. In addition, prolonged mechanical ventilatory duration is an independent risk factor for ventilator-induced lung injury in addition to oxygen toxicity, volutrauma, barotrauma, and atelectotrauma if not optimally managed [42, 43].

CDH patients with chronic PH have been shown to have longer durations of mechanical ventilation and hospital stay compared to those patients without chronic PH [8]. This mirrors what is seen in the PH associated with bronchopulmonary dysplasia [44, 45]. Separately, postnatally acquired infections can contribute to the development of chronic lung disease via gross inflammatory changes [46–49]. Amongst those neonates who required ECLS, Datta and colleagues identified a significant risk of developing chronic PH only in those who received high post-ECLS ventilator pressures, again suggesting a component of ventilator induced lung injury in the development of chronic PH [19].

While neonates with CDH will have catch-up lung growth, many continue to have abnormal lung function compared to healthy controls that likely mirror ongoing dysfunction of the pulmonary vasculature [50–52]. While Adaikalam and colleagues identified an increase in total lung volume post-CDH repair of 8 milliliters per week compared to fetal MRI lung volumes [53]; Koumbourlis and colleagues demonstrated that FRC, compliance, and resistance on pulmonary function tests were abnormal in infants with repaired CDH at 6 months of age [54]. Pulmonary function parameters remained abnormal at a mean age of 23 years, particularly in ECLS survivors [55]. Much less is known regarding the pulmonary vasculature over time.

Beyond primary lung etiologies, neonates with CDH often have longstanding feeding difficulties including slow feeding advancement, gastroesophageal reflux disease, and aspiration [56–59]. Chronic, repeated aspiration can cause inflammatory changes in the lung parenchyma resulting in lung injury, consolidation, and hypoxemia. These inflammatory changes can increase the susceptibility for the development of chronic PH [60, 61].

We hypothesize two natural history courses for this resistance-mediated phenotype. First, PVR is high at birth from typical fetal circulation but remains high secondary to abnormal pulmonary and pulmonary vascular development in fetuses with CDH. PVR then gradually falls with improvement in ventilation, cardiac support, and surgical repair to an endogenous nadir [27]. Then, with increasing post-natal insults that contribute to the development of chronic lung disease and vascular remodeling, PVR increases again leading to systemic or supra-systemic pulmonary pressures. Second, there is likely a group of neonates that never experience a complete nadir in fetal PVR and have persistent resistance-mediated chronic PH that is further exacerbated by remodeling and iatrogenic therapies. What exactly delineates these two courses of resistance-mediated PH is not yet known but could represent the contributions of the “two-hit” hypothesis. Further investigation is needed.

Treatment of resistance-mediated hypertension focuses on ventilation strategies to optimize FRC and ventilation-perfusion matching while minimizing lung overdistension and ventilator trauma, maintenance of normoxemia, avoidance of acidosis, and consideration of pulmonary vasodilator therapy which can include supplemental oxygen and pharmacologic vasodilators to lower PA pressures. While literature regarding the use of regarding pulmonary vasodilators for pulmonary arterial hypertension in adults and animal models has emerged favoring a potential vascular remodeling benefit [62], to date this effect has not been demonstrated in CDH or neonates in general. Optimization of postnatal somatic growth remains important. In neonates with proven aspiration, careful consideration should be given to feeding routes including transpyloric feeds and/or anti-reflux procedures such as Nissen fundoplication in the convalescent phase of care. Right heart cardiac catheterization should be considered in patients on a second-line pulmonary vasodilator therapy without improvement to confirm physiology and assess vasoreactivity.

### Flow-mediated PH (PAP = PBF x PVR + LAp)

Chronic PH can also develop from either absolute or relative excess PBF in neonates with CDH [63, 64]. This latter form originates when a hypoplastic pulmonary vascular bed is exposed to even normal blood flow, creating a relative excess in PBF. Echocardiographically, while this phenotype will demonstrate elevated pulmonary pressures (as in resistance-mediated), this phenotype can be distinguished by its markers of left-sided pressure-volume loading as well as cardiac output assessments [26].

Excess PBF can be derived from either iatrogenic pulmonary vasodilator therapy (including excessive FiO_2_ contributing to supra-physiologic PaO_2_) or persistent left-to-right shunts (e.g. PDA, ventricular septal defects (VSD), atrial septal defects (ASD), or other arteriovenous connections) [65–67]. Reflecting this, a prospective cohort study by Kraemer and colleagues showed that CDH patients with chronic PH were more likely to be treated with pulmonary vasodilators during their initial hospital admission compared to patients without chronic PH [8].

The persistence of fetal shunts, specifically the PDA, is a complicating factor in the care of neonates with CDH and can be a source of excessive PBF. In the acute period, it can be beneficial by supporting pulmonary blood flow in the setting of severe right ventricular (RV) dysfunction or RV function in the setting of suprasystemic PH. Similarly, during severe left ventricular (LV) dysfunction or in cases of left-heart obstruction more common in neonates with CDH (aortic coarctation, severe arch hypoplasia), the PDA can support systemic blood flow [68]. Due to these early effects, pharmacologic maintenance of the PDA with prostaglandins is increasingly utilized in neonates with CDH [69]. However, these beneficial effects may be time limited. As cardiac function improves and PVR decreases to sub-systemic levels, PDA shunt volume increases, mirroring preterm PDA physiology [65]. Clinically, recognizing this physiologic transition is subtle yet crucial to inform bedside management, specifically regarding prostaglandin infusions. This can present as a patient with worsening oxygenation and ventilation without a pre- and post- ductal saturation differential.

Other intracardiac shunts, including large ASDs and/or VSDs, are important considerations as these left-to-right shunts can also contribute to excess PBF [70]. Arteriovenous malformations, similarly, though rare, are classic examples of excess venous return to the right atrium and flow-mediated pulmonary hypertension. A comprehensive hemodynamic and echocardiographic evaluation that includes cardiac output measurements can signal to the clinician the need to modulate these shunts.

We hypothesize that the natural history of the flow-mediated phenotype is time-limited and is followed by a resistance-mediated phenotype. As PVR falls, pathologic shunts will flow persistently left-to-right, and the continued iatrogenic use of pulmonary vasodilators and prostaglandins will further increase excess pulmonary blood flow, leading to an increase in PAP via this flow-mediated phenotype. Over time, with sustained excess PBF, vascular remodeling occurs due to endothelial dysfunction, smooth muscle cell proliferation, and extracellular matrix deposition which results in an increase in PVR and transition back to a resistance mediated phenotype (an Eisenmenger-type phenomenon) [71, 72]. It is important to understand the temporal relationships of these phenotypes as this dynamic nature informs management strategies.

Treatment of flow-mediated chronic PH includes shunt mitigation strategies and diuretics. Of note, due to its association with renal release of endogenous Prostaglandin E2 [73], furosemide should be used cautiously in the presence of a PDA. Shunt mitigation strategies include optimizing pH, PaO_2_, PaCO_2_, serum hemoglobin, and platelet levels in addition to consideration of pharmacologic (acetaminophen, non-steroidal medication) or definitive operative or endovascular shunt closure. Notably, neonates with a flow-mediated chronic PH phenotype can be harmed using pulmonary vasodilators and prostaglandin infusions, highlighting the importance of phenotypic characterization.

### Post-capillary PH (PAP = PBF x PVR + LAp)

Post-capillary chronic PH is likely an under-recognized phenotype in the CDH population. Left heart hypoplasia is a known acute feature of both left- and right-sided CDH and is associated with adverse outcomes[74]. Less is known regarding the changes in left heart structure over time, though Vogel and colleagues in a single-center study reported gradual normalization of left-heart structure z-scores over time post-repair [75]. It is plausible that left heart growth exists on a spectrum, and some neonates may have more persistent LV hypoplasia predisposing to post-capillary PH, though evidence in this domain is currently lacking. Cardiac dysfunction in CDH is typically transient, but in some neonates can continue into infancy and even childhood, especially LV diastolic dysfunction (LVDD) [76–78]. LVDD was shown to be present in approximately 50% of neonates with CDH who underwent cardiac catheterization at a mean age of 78 post-natal days [77]. Etiologies of LVDD include unrecognized systemic hypertension, medication side effects, and underlying genetic predisposition. Additionally, the frequent use of systemic corticosteroids has the potential to cause both systemic hypertension and LVDD [79]. More research is needed to understand the chronic implications of diastolic dysfunction in this population.

In addition, there is a small but growing appreciation for the potential role of pulmonary vein disease in children with a history of CDH. Mechanistically, this is predicted secondary to a “no flow, no grow” paradigm in which pulmonary veins, particularly on the ipsilateral side of the CDH, may be hypoplastic and prone to developing stenosis post-natally. A small study by Kinsella and colleagues demonstrated pulmonary vein stenosis or delayed pulmonary venous return in 6 out of 7 patients with CDH and chronic PH who underwent cardiac catheterization [80].

Cumulatively, these factors play a role in the development of a post-capillary chronic PH phenotype, characterized by elevated left atrial pressure (excluding pulmonary vein disease), pulmonary venous hypertension, and upstream elevated PAP. In the case of isolated pulmonary vein disease, LA pressure may not be elevated while the pulmonary capillary wedge pressure (PCWP) may be elevated on right heart catheterization. The natural history of post-capillary chronic PH in neonates with CDH is likely variable depending on the underlying etiology. This is a challenging diagnosis to make, and if suspected, would warrant consideration of cardiac catheterization to fully delineate the underlying physiology. The prognosis is variable; primary pulmonary vein disease causes persistent and gradually worsening symptoms while LVDD likely has a waxing and waning course depending on cardiac loading conditions. Both cause elevated PAP. However, the key driver of LVDD is elevated LV end-diastolic pressure which is not seen in pulmonary vein disease patients.

Small but significant natural history studies suggest the cardiac maldevelopment that occurs *in utero* continues to impact cardiac performance into at least childhood and potentially beyond. Egan and colleagues identified reduced RV systolic and diastolic metrics (by tissue Doppler imaging) in seven children with a history of CDH compared to similarly aged controls at 6 years of age [81]. Similarly, Abolmaali and colleagues found reduced biventricular stroke volume using cardiac MRI in 12-year-old children with a history of CDH compared to non-CDH controls [82].

Treatment of post-capillary chronic PH is multifactorial and can include the use of anti-hypertensive medications for afterload reduction in the setting of systemic hypertension. In the acute setting, milrinone can be used to augment afterload reduction and cardiac function, but it is limited by its intravenous nature. It should be used with caution in patients with renal impairment. Chronically, angiotensin-converting enzyme inhibitor (ACEi) therapy, which has been shown to be safe and feasible in BPD-associated PH [83], is an option for neonates with signs of LVDD and systemic hypertension. Diuretics can decrease LA hypertension (or in the case of pulmonary vein disease, pulmonary venous congestion) and provide symptomatic improvement. Pulmonary vein disease should be evaluated for procedural intervention depending on severity. Similar to flow-mediated PH, pulmonary vasodilator therapy is contraindicated in this population.

### Mixed phenotypes

Some neonates with CDH can demonstrate mixed PH phenotypes and may require a combination therapy approach (e.g. low-dose pulmonary vasodilator and diuretic therapy). Examples of these mixed phenotypes include the overlapping transition from flow-mediated to resistance-mediated chronic PH in the setting of vascular remodeling (discussed above) as well as the potential co-existence of a post-capillary phenotype with an intrinsic resistance-mediated process either from vascular remodeling or native pulmonary vascular hypoplasia. The overlap of chronic PH phenotypes and the distinct, complex physiology of these patients emphasize the need for serial, comprehensive and multiparametric echocardiographic (and potentially cardiac catheterization) assessments to fully understand each component of chronic PH in these patients.

### Assessing phenotypes

We recommend comprehensive echocardiography and hemodynamic assessment to delineate the predominant underlying phenotype prior to discharge (ideally within two weeks). This can help guide initial outpatient management. Recognition that these phenotypes can transition over time and with medication adjustments; however, ideally suggests a reassessment of the predominant phenotype at *each* echocardiogram post-discharge. At the least, we recommend a comprehensive phenotype reassessment within 6 months of initial discharge if the infant remains on oxygen and/or PH medications. This should include consideration of right heart catheterization if phenotype assessment is unclear or clinical progress has not been made, as is described below.

## Proposed screening and follow up of chronic pulmonary hypertension in neonates with CDH

**Fig. 2:**
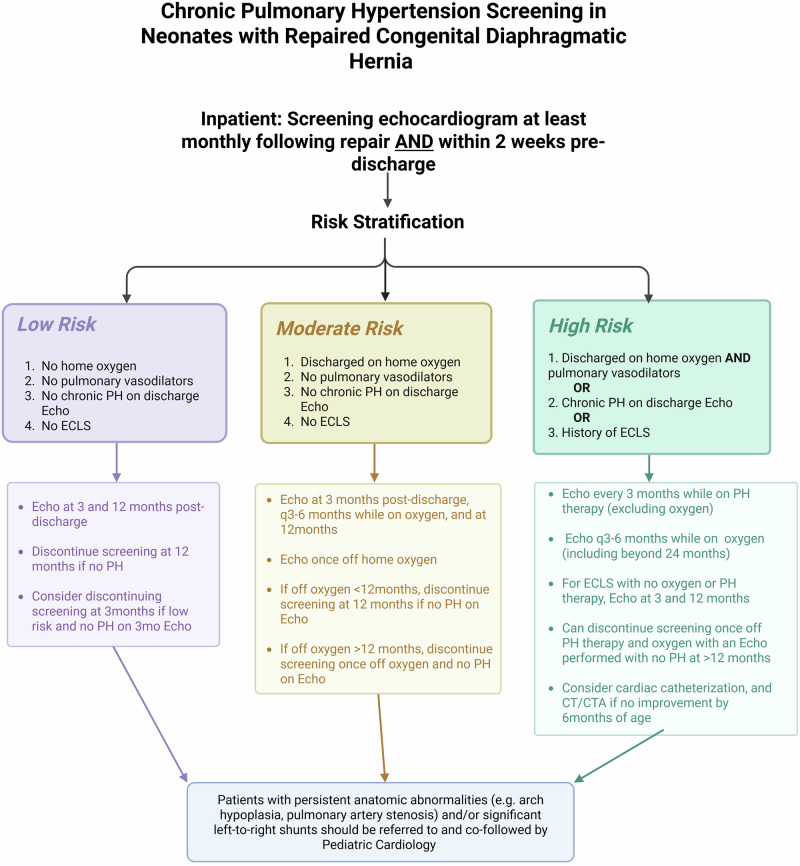
Proposed outpatient screening algorithm for chronic pulmonary hypertension in neonates with congenital diaphragmatic hernia. Screening frequency is determined by risk stratification based upon echocardiographic criteria and clinical history.

There are no formal recommendations on timing of surveillance screening of chronic PH in neonates with CDH. Recently, Cimbak and Buchmiller published a proposed comprehensive long-term follow-up regimen for patients with CDH that included screening echocardiograms at regular intervals [84]. Based on this review and institution-specific protocol, we propose a risk-stratified approach to chronic PH screening in neonates and children with CDH shown in Fig. 2.

## Knowledge gaps and areas of future studies

There remains a paucity of evidence on many facets of chronic PH in CDH. More research is needed to understand the natural history of chronic PH in this population, which would inform recommendations on the timing of chronic PH surveillance. Specifically, LVDD in CDH is not well-studied, and more research is needed to better understand and treat this population. Further, the growing use of prostaglandin therapy will need to be observed and investigated carefully regarding its potential for contributing to chronic PH development. The increasing use of genetic studies in neonates with CDH may lead to a greater understanding regarding each patient’s risk predisposition. Standardized frameworks for imaging protocols for neonates with CDH are necessary to collaborate across institutions and improve our ability to study this group in addition to the creation of echocardiography registries to centralize data for future research.

## Conclusion

Chronic PH in neonates with CDH is an understudied disease process. The importance of physiology-targeted care instead of symptom-based management cannot be understated as different physiologies mandate different, and often times, opposing treatments. Serial, multi-parametric hemodynamic assessments that allow for real-time interpretation and management of chronic PH in CDH patients are crucial to optimize care of these neonates. Further investigation is needed to better understand the natural history of chronic PH in CDH, the prevalence of each phenotype, to evaluate early risk factors for future development of chronic PH and to determine optimal treatment of chronic PH in this complex disease.
